# Relationship between Oxidative Stress and Imatinib Resistance in Model Chronic Myeloid Leukemia Cells

**DOI:** 10.3390/biom11040610

**Published:** 2021-04-20

**Authors:** Sylwester Głowacki, Ewelina Synowiec, Marzena Szwed, Monika Toma, Tomasz Skorski, Tomasz Śliwiński

**Affiliations:** 1 Laboratory of Medical Genetics, Faculty of Biology and Environmental Protection, University of Lodz, Pomorska 141/143 Street, 90-236 Lodz, Poland; sylwester.glowacki@me.com (S.G.); ewelina.synowiec@biol.uni.lodz.pl (E.S.); monika.toma@biol.uni.lodz.pl (M.T.); 2 Department of Medical Biophysics, Faculty of Biology and Environmental Protection, University of Lodz, Pomorska 141/143 Street, 90-236 Lodz, Poland; marzena.szwed@biol.uni.lodz.pl; 3 Fels Cancer Institute for Personalized Medicine, Temple University Lewis Katz School of Medicine, Philadelphia, PA 19140, USA; tskorski@temple.edu

**Keywords:** chronic myeloid leukemia, imatinib, reactive oxygen species

## Abstract

Chronic myeloid leukemia (CML) develops due to the presence of the BCR-ABL1 protein, a target of tyrosine kinase inhibitors (TKIs), such as imatinib (IM), used in a CML therapy. CML eradication is a challenge due to developing resistance to TKIs. BCR-ABL1 induces endogenous oxidative stress leading to genomic instability and development of TKI resistance. Model CML cells susceptible or resistant to IM, as well as wild-type, non-cancer cells without the BCR-ABL1 protein were treated with IM, hydrogen peroxide (H_2_O_2_) as a model trigger of external oxidative stress, or with IM+H_2_O_2_. Accumulation of reactive oxygen species (ROS), DNA damage, activity of selected antioxidant enzymes and glutathione (GSH), and mitochondrial potential (MMP) were assessed. We observed increase in ROS accumulation in BCR-ABL1 positive cells and distinct levels of ROS accumulation in IM-susceptible cells when compared to IM-resistant ones, as well as increased DNA damage caused by IM action in sensitive cells. Depletion of GSH levels and a decreased activity of glutathione peroxidase (GPx) in the presence of IM was higher in the cells susceptible to IM. IM-resistant cells showed an increase of catalase activity and a depletion of MMP. BCR-ABL1 kinase alters ROS metabolism, and IM resistance is accompanied by the changes in activity of GPx, catalase, and alterations in MMP.

## 1. Introduction

Chronic myeloid leukemia (CML) is a result of an exchanged translocation between chromosomes 9 and 22, t(9;22)(q34;q11), which leads to the formation of the Philadelphia (Ph) chromosome harboring fusion oncogene BCR-ABL1 [1]. The BCR-ABL1 protein expressed from this gene is a constitutively active tyrosine kinase that confers growth advantages to Ph-positive cells through disturbances in multiple signaling pathways, including those involved in controlling cellular growth or apoptosis [2,3]

There are several pharmacological agents targeting BCR-ABL1 kinase developed during recent years. IM which acts as a TKI, was the first among them. During chemotherapy cells exposed to IM undergo rapid apoptosis, that in most patients manifests itself in the form of a complete, hematologic response (reviewed in [4]). However, some leukemic cells from the hematopoietic stem cell population are resistant to IM therapy and their survival does not seem to be dependent on BCR-ABL1 activity. They act as a reservoir from which mutated clones of cells fully resistant to IM can appear at some point leading to a relapse of the disease [5]. This phenomenon is known as acquired or secondary resistance, and point mutations in BCR-ABL1 were established as a main cause of it [3]. Different mechanisms may be the basis of primary resistance that reveals itself in a small cohort of IM-naive patients who are unresponsive to IM treatment even at the outset of therapy, including the action of cellular influx and efflux transporters. [6].

Recent studies point to conclusions that multiple factors can influence and contribute to the development of resistance to TKIs such as IM. These may include susceptibility of BCR-ABL1-positive cells to diverse proapoptotic signals including ROS or other secondary messengers involved in the mitochondrial metabolism.

Apoptosis usually starts through two main pathways: the death receptor- or mitochondria-mediated pathway, also known as the extrinsic and intrinsic pathway, respectively [7]. In both pathways, ROS are the crucial modulators that affect signaling cascades involved in apoptosis regulation. While ROS act as redox messengers at physiological levels, their elevated activity is usually associated with the programmed cell death induction [8]. Moreover, it is well established that cellular redox status is one of the most fundamental factors regulating mitochondrial membrane permeabilization. As such, ROS may also mediate in the release of cytochrome C from mitochondrial matrix and through a depletion of mitochondrial membrane potential trigger apoptosis [9].

It has been shown that BCR-ABL1 cells have elevated levels of ROS indicating increased oxidative stress and its role in leukemia’s progression [10,11,12,13,14,15,16]. Some other cellular sources of ROS were also implicated in pathogenesis of CML, including those generated through NADPH oxidase (NOX) activity [17,18,19]. Hyperactivation of the FLT3/ITD oncokinase triggers increase in the transcription for *NOX* gene [20]. Interestingly, about 30% of leukemia patients show internal tandem duplications in the FMS-like tyrosine kinase-3 (FLT3) receptor, which controls the expression of the mentioned enzyme [21]. The influence of the oxidative and nitrosative stress on CML progression is not clear. It was confirmed that the overproduction of free radicals in the expansion of leukemia, as well as the exogenous ROS and NOS (nitrogen species) generation can be related with the cellular resistance to IM [22]. The broad extensive oxidative and nitrosative stress exert genotoxic effects on cancer cells and mediate the induction of programmed cell death [23].

Therefore, the understanding of the interaction of ROS signaling and BCR-ABL1 activity is of utmost importance [24,25].

In this study we intended to determine whether 32D cells susceptible and resistant to IM differ in physiological and induced levels of ROS generation, the amount of basal and induced oxidative DNA damage, the total level, and the reduced and oxidized ratio of glutathione (GSH), the activity of ROS-detoxifying enzymes and the relation between mitochondrial potential and resistance to IM.

## 2. Materials and Methods

### 2.1. Chemicals

IM was provided by Novartis. IM was prepared as a stock of 10 mM concentration in DMSO further diluted to target concentrations with a medium before the experiment. The IMDM medium was purchased from Gibco BRL, the DCFH-DA probe was obtained from Sigma Chemicals, the Glutathione Peroxidase Assay Kit was obtained from Cayman Chemical, the OxiSelect^TM^ Superoxide Dismutase Activity Assay and OxiSelect^TM^ Catalase Activity Assay Kit, Colorimetric were obtained from Cell Biolabs and the GSH/GSSG Ratio Detection Assay Kit (Fluorometric–Green) was obtained from Abcam.

### 2.2. Cells

Murine 32D clone 3 cell lines: 32D BCR-ABL1 sensitive to IM (32D BA S), 32D BCR-ABL1 Y253H primarily resistant to IM (32D BA R) and 32D cells without the BCR-ABL1 oncogene (32D P) were used in this study. The 32D Clone 3 (ATCC^®^ CRL-11346™) cells were obtained from American Type Culture Collection (ATCC) and transfected with BCR-ABL1, wild type or with Y253H mutation as described previously [26]. Additionally, the 32D BCR-ABL1 with acquired resistance to IM (32D BA IM-AR) was created in-house by growing the 32D BA IM-S cells at increasing concentrations of IM (Novartis, Basel, Switzerland), from 0.05 up to 1 µM.

The cells were cultured in the IMDM medium (Lonza, Basel, Switzerland) supplemented with 2 mM L-glutamine, 100 U/mL penicillin, 100 μg/mL streptomycin, 10% fetal bovine serum and kept in an incubator with 5% CO2 atmosphere at 100% humidity and 37 °C. The 32D P line needed additional supplementation with interleukin 3 to proliferate, which was delivered through the addition of filtered supernatant produced by WEHI-3B cells (15% conditioned medium) as described previously [27]. WEHI-3B conditioned media containing IL-3 was produced by culturing the WEHI 3B cells to a high density and then harvesting the supernatants.

### 2.3. MTT Assay for Cell Viability

Viability of the cells treated with imatinib was assessed with the MTT assay as described previously [16]. Briefly: cells were cultured in a 96-well plate at 37 °C and exposed to different concentrations of imatinib (0.01–1 μM) for 24 h or H_2_O_2_ (100 μM) for 0.5 h. As negative controls we used cells treated solely with culture. Cells were then washed with PBS and incubated with thiazolyl blue tetrazolium bromide (MTT) solution. MTT was then discarded carefully, and dimethyl sulfoxide (DMSO) was added to resolve the formazan crystals. Microplate Reader 550 (Bio-Rad Laboratories, Hercules, CA, USA) was used to measure absorbance in each well at 595 nm with a reference wavelength of 655 nm. Ratio of absorbances of treated to untreated living cells was used as measure of viability.

### 2.4. Detection of BCR-ABL1 Presence

The presence of Bcr-Abl1 was determined using PathScan Phospho-Bcr-Abl (Tyr177) Sandwich ELISA (Cell Signaling, Beverly, MA, USA), according to the manufacturer’s instructions. Briefly, cells were lysed in 1 mL of cell lysis buffer (provided as a kit component). A normalized amount of protein was loaded into each well, which was then sealed with a tape and incubated for 2 h at 37 °C. After four washes in wash buffer (provided as a kit component) detection antibody reagent were added to each well and incubated for 1 h at 37 °C. The washing procedure was repeated and reconstituted HRP-linked secondary antibody was added and incubated for 30 min at 37 °C. Later, TMB substrate was added and incubated for 10 min at 37 °C. Finally, the reaction was terminated with a STOP solution and plates were read by measuring absorbance at 450 nm.

The presence of Bcr-Abl was also confirmed through a quantitative real-time PCR analysis (mRNA *BCR-ABL1* level). Total RNA was extracted from 5 × 10^6^ cells using ISOLATE II RNA Mini Kit (Bioline Reagents Ltd., London, UK). Next, first-strand cDNA was synthesized from total RNA using High-Capacity cDNA Reverse Transcription Kit (Thermo Fisher Scientific, Waltham, MA, USA). Then, 2 ng of total RNA was used as a template in a total volume of 20 µL. *BCR-ABL1* expression was analyzed by using the TaqMan^®^ primer and probe set Hs03024784_ft and TaqMan™ Universal Master Mix (Thermo Fisher Scientific). The reactions were carried out in a thermal cycler CFX96 ™ Real-Time PCR Detection System (BIO-RAD Laboratories, Hercules, CA, USA). The thermal cycling conditions were as follows: 10 min of polymerase activation at 95 °C, followed by 40 cycles of 30 s denaturation at 95 °C and 60 s annealing/extension at 60 °C. Each sample was run in triplicate. The cycle threshold (Ct) values were calculated automatically by CFX96 ™ Real-Time PCR Detection System (BIO-RAD) software and the expression level was calculated using the 2^−ΔCt^ model, with the Ct values normalized using 18S rRNA as internal (endogenous) control.

### 2.5. Detection of BCR-ABL Y253H Mutation

Mutations of the BCR-ABL1 Y253H in 32D cells were analyzed by allele-specific real-time PCR (AS RT-PCR) method. Total RNA was extracted from 1 × 10^6^ cells by using the ISOLATE II RNA Mini Kit (Bioline Reagents Ltd.; London, UK) following the manufacturer’s instructions. Once extracted, RNA concentration and purity were verified by UV measurement, using the Bio-Tek Synergy HT Microplate Reader (Bio-Tek Instruments, Winooski, VT, USA). Subsequently, single-stranded cDNA was synthesized from 2 μg of total RNAs using the High-Capacity cDNA Reverse Transcription Kit (Applied Biosystems™). For Y253H mutation detection, the following primer sequences were used: forward 5′-ACTCCAGACTGTCCACAGCAT-3′ and allele specific reverse 5′-CGTACACCTCCCCGTG-3′. Thermal cycling conditions for allele-specific PCR amplifications were optimized on the CFX96™ Real-Time PCR Detection System (BIO-RAD) and the results were analyzed by using CFX Manager™ Software version 3.1 and gel electrophoresis. The AS RT-PCR conditions were as follows: UDG pre-treatment at 50 °C for 2 min, an initial denaturation step for 10 min at 95 °C, followed by up to 50 cycles at 95 °C for 15 s, 60 s at 60 °C annealing/extension with plate reading at this step. Reaction mix consisted of 1 × Luminaris HiGreen qPCR Master Mix (Thermo Fisher Scientific, West Palm Beach, FL, USA), including SYBR Green I fluorescent dye, 0.3 µM each primer (Sigma-Aldrich, Hamburg, Germany) and 1 μL of cDNA in a total volume of 10 µl per sample. The cycle threshold (Ct) values were calculated automatically by CFX96™ Real-Time PCR Detection System software (BIO-RAD). The basal expression level was calculated using the 2^−ΔCt^ model with ACTB as an internal control.

### 2.6. Assessment of ROS Level

The ROS level was evaluated with OxiSelect Intracellular ROS Assay Kit (Cell Biolabs, San Diego, CA, USA). Briefly: cells were cultured in a 96-well plate at 37 °C and exposed to different concentrations of imatinib (0.01–1 μM) for 24 h or H_2_O_2_ (100 μM) for 0.5 h, washed, and then treated according to the kit manufacturer’s protocol. This cell-based assay is applied for measuring hydroxyl, peroxyl, or other reactive oxygen species activity within a cell, which employs the cell-permeable fluorogenic probe 2′,7′-dichlorodihydrofluorescein diacetate. In short, DCFH-DA is diffused into cells and is deacetylated by cellular esterase to non-fluorescent 2′,7′- dichlorodihydrofluorescein, which is rapidly oxidized by ROS to highly fluorescent 2′,7′- dichlorodihydrofluorescein. The fluorescence intensity is proportional to the ROS levels within the cell cytosol. The data obtained were presented as mean ± S.E.M. from three experiments.

### 2.7. Assessment of DNA Damage and Repair

The comet assay was performed in the alkaline version (pH 13) as described previously [28]. The slides with processed cells were examined at 200× magnification in an Eclipse fluorescence microscope (Nikon, Tokyo, Japan) attached to a COHU 4910 video camera (Cohu, San Diego, CA, USA) equipped with a UV-1 filter block consisting of an excitation filter (359 nm) and a barrier filter (461 nm) and connected to a personal computer-based image analysis system—Lucia-Comet v. 5.41 (Laboratory Imaging, Praha, Czech Republic).

Around fifty images were randomly selected from each sample and the percentage of DNA in the tail of the comets (% tail DNA) was measured (the quantity varied in the case of some samples). The mean value of the % tail DNA in a particular sample was taken as an index of DNA damage in the specific sample. All experiments were performed in duplicate. The data obtained in the experiments on DNA damage and repair were expressed as means with SEM from three experiments.

### 2.8. Cell Extracts

To assess the activity of selected enzymes active in ROS metabolism, as well as GSH levels and GSH to GSSG ratios, cell lysate was first prepared. Eight million cells were gathered after previously conducted counting in a Bürker chamber to get 1 mL of cell lysate. Cells were centrifuged in 1500× *g* in 4 °C for 5 min. The pellet was washed with PBS, cooled on ice, and then suspended in a lysis buffer consisting of 10 mM Tris, pH 7.5, 150 mM NaCl and 0.1 mM EDTA. The cells were lysed sonically (30% amplitude, 20 s single impulse, 5 s breaks between impulses, repeated three times) on ice. After that, the lysate was centrifuged in 4 °C for 10 min in 10,000× *g*, the pellet was discarded, and the lysate was frozen in −80 °C.

### 2.9. Assessment of GSH/GSSG Levels

To assess cellular GSH and GSSG levels and their ratio fluorometric GSH/GSSG Ratio Detection Assay Kit (Abcam, Cambridge, UK) was used according to the manufacturer’s protocol with lysate obtained as described above. Briefly, the sample cell lysate or GSH, or GSSG standards were added to the thiol green indicator reaction mixture and were incubated for 60 min in room temperature and protected from light. Then an increase in fluorescence was monitored at Ex/Em = 490/520 nm with Bio-Tek Synergy HT Microplate Reader (Bio-Tek Instruments, Winooski, VT, USA). GSH and GSSG standards were used to create a graph of the standard curve. Fluorescence in blank wells (with the assay buffer only) was used as control and was subtracted from the values for those wells with the GSH reactions. Reduced GSH and total GSH were measured through calculation form standard curves and the GSSG amount was calculated as a difference between total GSH and reduced GSH.

### 2.10. Activity of Glutathione Peroxidase

We measured the activity of GPx with the Glutathione Peroxidase Cellular Activity Assay Kit (Sigma-Aldrich, St. Louis, MO, USA) according to the manufacturer’s protocol using lysate obtained as described in 2.8. This kit applies an indirect determination method. It is based on the oxidation of GSH to GSSG catalyzed by GPx, which is then coupled to the recycling of GSSG back to GSH utilizing glutathione reductase and NADPH. We measured the decrease in NADPH absorbance at 340 nm with the Bio-Tek Synergy HT Microplate Reader (Bio-Tek Instruments, Winooski, VT, USA), during the oxidation of NADPH to NADP+ acting as indicative of GPx activity, since GPx was the rate limiting factor of the coupled reactions.

### 2.11. Activity of Catalase

Evaluation of catalase activity was performed with the Catalase Assay Kit (Sigma-Aldrich, St. Louis, MO, USA) according to the manufacturer’s protocol using lysate obtained as described in 2.8. This assay involved measuring the H_2_O_2_ substrate remaining after the action of catalase. Catalase converted H_2_O_2_ to water and oxygen, and the reaction was then stopped with sodium azide. An aliquot of the reaction mix was assayed for the amount of H_2_O_2_ remaining by a colorimetric method with the Bio-Tek Synergy HT Microplate Reader (Bio-Tek Instruments, Winooski, VT, USA), where the substituted phenol (3,5-dichloro-2-hydroxybenzene- sulfonic acid) was coupled oxidatively to 4-aminoantipyrine in the presence of hydrogen peroxide and horseradish peroxidase (HRP) to give a red quinonimine dye (*N*-(4-antipyryl)-3-chloro-5-sulfonate-p-benzoquinone-monoimine) that absorbs at 520 nm.

### 2.12. Mitochondrial Membrane Potential

MMP was assessed using the MitoProbe JC-1 Assay Kit (Life Technologies). The kit includes the cationic dye JC-1 (5′,6,6′-tetrachloro-1,1′,3,3′-tetraethylbenzimidazolylcarbocyanine iodide) together with the mitochondrial membrane potential disrupter CCCP (carbonyl cyanide 3-chlorophenylhydrazone). Carbocyanine dye accumulates in the mitochondrial membrane in a potential-dependent manner. A high potential of the inner mitochondrial membrane leads to the formation of dye aggregates with both excitation and emission shifted towards red light (530 nm/590 nm), while respective values for JC-1 monomers are 485 nm/538 nm. Then, 5 × 10^4^ cells in 100 µL culture medium, with or without tested agents, were seeded into plate wells and cultured at 37 °C for 24 h in CO_2_-containing environment. Then, the cells were incubated with 5 μM JC-1 in HBSS in CO_2_ atmosphere at 37 °C for 30 min, centrifuged (300× *g* for 10 min at 22 °C) and then washed twice with HBSS. Bio-Tek Synergy HT Microplate Reader (Bio-Tek Instruments, Winooski, VT, USA) with filter pairs of 530 nm/590 nm and 485 nm/538 nm was used for fluorescence measurements. The results were shown as a ratio of aggregates to monomer fluorescence.

### 2.13. Statistical Analyses

All statistical analyses of differences between lines in different treatments were performed in Prism 6 software (GraphPadSoftware, Inc., San Diego, CA, USA) using ordinary one-way ANOVA followed with Dunnett’s multiple comparison test. Results were expressed as means with SEM. The experiments were performed in triplicate, *n* ≥ 3.

## 3. Results

### 3.1. Detection of BCR-ABL1 Presence and Its Mutational Status

**Figure 1:**
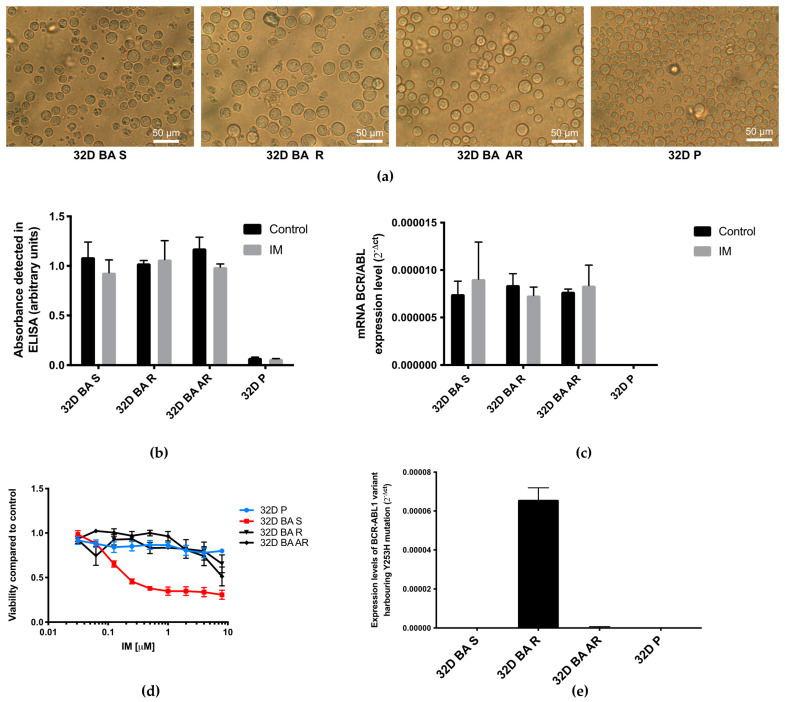
(**a**) Morphology of 32D cells, 32D cells transfected with BCR-ABL1 oncogene susceptible to tyrosine kinase inhibitors (32D BA S), cells transfected with BCR-ABL1 oncogene harboring Y253H point mutation that exhibited primary resistance to IM (32D BA R) and 32D cells transfected with BCR-ABL1 oncogene and cultured in increasing concentration of IM until they developed acquired IM-resistance (32D BA AR line), and wild type (32D P). (**b**) Presence of BCR-ABL1 protein detected by ELISA test; (**c**) Analysis of BCR-ABL1 expression normalized to 18S rRNA as internal (endogenous) control; (**d**) The viability curves of leukemia cells treated with IM. (**e**) The mutational analysis of 32D cells for Y253H mutation in *BCR-ABL1* gene. 32D BA S cells were compared to 32D BA R and 32D BA AR cells. 32D BA P cells were used as additional control. IM was used in 1 μM concentration for 24 h (**b**,**c**) or in various concentrations of up to 10 μM for 24 h (**d**). We presented results as mean ± SEM.

We started with a verification of the presence of BCR-ABL1 fusion gene product in cells susceptible to IM (32D BA S), primary resistant to IM due to Y253H mutation (32D BA R), and with acquired resistance, developed through culturing the cells in medium with an increasing IM concentration (32D BA AR). As the control cells, we used wildtype 32D P cells without BCR-ABL1 gene resent. The examined cells differed in their size, shape, and homogeneity (Figure 1a). Interestingly, the BCR-ABL1-positive cells were characterized by a bigger diameter and less homogenous population suggesting that oncogenic transformation influenced their phenotype. Figure 1-b shows that the product of the BCR-ABL1 expression remained present in the examined cells even after IM treatment and 1-c confirms this for normalized expression levels. This allowed us to confirm that all the observed effects were due to alteration in downstream signaling, not the lack of expression of the BCR-ABL1 protein in cells treated with IM. As is shown in Figure 1d, the studied cell lines exhibited significantly different viability after IM-treatment.

We also investigated the status of Y253H mutation (Figure 1e) and we detected it clearly in 32D BA R cell line, which is specifically transformed with variant of BCR-ABL1 gene harboring that mutation.

### 3.2. Reactive Oxygen Species Levels Are Increased in BCR-ABL1 Positive Cells Susceptible to IM When Compared to BCR-ABL1 Negative Cells, and Are Lower in Cells with IM-Acquired Resistance after Treatment with IM

**Figure 2:**
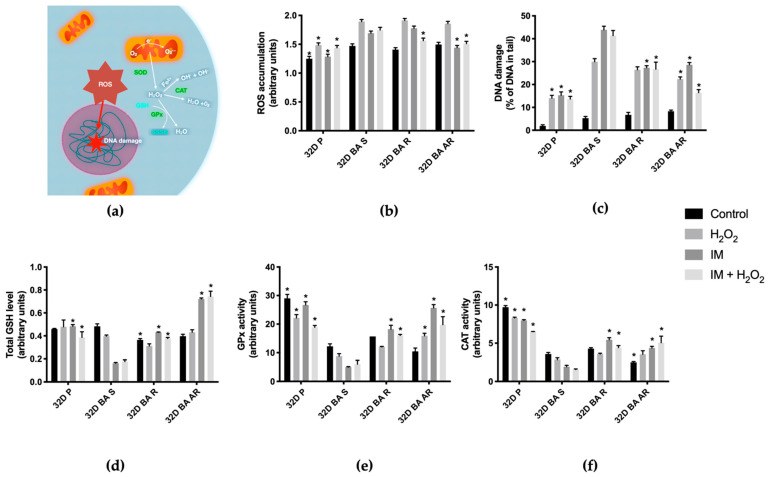
(**a**) Schematic overview of potential oxidative stress responses that may be induced in leukemia cells. The production of ROS is triggered by the disturbances of mitochondria function and causes the activation of antioxidant enzymes or cellular damage, e.g., DNA lesions; (**b**) Comparison of the level of reactive oxygen species in BCR-ABL1—positive and parental 32D P cells under control conditions and after treatment with IM, H_2_O_2_ or IM + H_2_O_2_; (**c**) Comparison of DNA damage in BCR-ABL1—positive and parental 32D P cells under conditions as described above; (**d**) Comparison of total GSH level in BCR-ABL1—positive and parental 32D cells under conditions as described above (**e**) Comparison of glutathione peroxidase activity in BCR-ABL1—positive and parental 32D cells under conditions as described above; (**f**) Comparison of catalase activity in BCR-ABL1—positive and parental 32D cells under conditions as described above. We cultured cells with IM for 24 h and with H_2_O_2_ for 30 min; in case of co-incubation, we added H_2_O_2_ at the end of IM incubation. IM was used in 1 μM concentration for 24 h and H_2_O_2_ in 100 μM for 30 min. * *p* < 0.05, the asterisk indicates a significant difference after treatment between a given cell line and the 32D BA S cells, *n* ≥ 3, we presented results as mean ± SEM.

Basal levels of ROS production seemed similar in all BCR-ABL1 positive cells, both susceptible and resistant to IM action. The only statistically significant difference was noted between those cells and non-leukemic 32D P cells, with the latter having lower levels of detected ROS (Figure 2b).

This pattern repeats after 24-h treatment with H_2_O_2_, which we added to simulate conditions of exogenous oxidative stress. After exposition to IM 32D BA AR and 32D P cell lines exhibited a significantly lower level of ROS accumulation when compared to the 32D BA S line, while the 32D BA R line still seemed to show the level of ROS production, as high as in the case of the 32D BA S line.

Differences were most pronounced after co-incubation with IM and H_2_O_2_, when 32D BA S line accumulated significantly more ROS than all other lines.

### 3.3. Exposure to IM and/or H_2_O_2_ Increases DNA Damage in IM-Susceptible Cells

We examined DNA damage levels in BCR-ABL1 positive and negative cells using a comet assay (Figure 2c).

We noticed that there was no significant difference in the basal levels of DNA damage between the 32D BA S line and the remaining investigated cells. Treatment with H_2_O_2_ changed the situation. The 32D P and BCR-ABL1-positive IM-resistant cells showed the lowest level of accumulated DNA damage, albeit the difference between the 32D BA S line and the 32D BA R cells was not statistically significant. Treatment with IM or IM+ H_2_O_2_ exacerbated those differences and the 32D BA S line accumulated significantly more DNA damage than other lines.

### 3.4. GSH Level Decreases in IM-Susceptible Cells after Exposure to IM When Compared to IM-Resistant Cells

Glutathione is an antioxidant that exists in either reduced (GSH) or oxidized (GSSG) form and prevents oxidative damage to various cellular structures. The ratio of GSH to GSSG can be used as a measure of oxidative stress in cells [29].

GSH level (Figure 2d) under control conditions was lower in the cells resistant to IM and P-line cells, compared to the S-line cells; however, the difference was not significant between the 32D BA S and the 32D P line. We detected no significant differences after treatment with H_2_O_2_. Following the incubation with IM alone or IM in combination with H_2_O_2_, the GSH level was significantly lower in 32D BA S cells in comparison to other lines. Interestingly, we did not detect any significant differences of the GSH to GSSG + GSH ratio between the investigated cell lines (data not shown).

### 3.5. Enzymes Involved in ROS Metabolism Are More Active in IM-resistant Cells after Exposure to IM

We analyzed the activity of selected enzymes involved in oxidative balance regulation to get a better picture of the antioxidant defenses in IM-susceptible and resistant cells.

#### 3.5.1. Glutathione Peroxidase

GPx is a family of peroxidases catalyzing the reaction of H_2_O_2_ reduction to water by GSH. As such, they play an essential role in the regulation of ROS-signaling. Recently, their role in carcinogenesis has also been extensively discussed [30].

We observed that the 32D BA S line always exhibited a lower level of GPx activity than the 32D P line. However, after IM treatment and incubation in parallel with IM and H_2_O_2_, 32D BA S cells had also a significantly lower GPx activity than the IM-resistant ones (Figure 2e).

#### 3.5.2. Catalase

Catalase catalyzes the reduction of hydrogen peroxide to water and oxygen and is an essential element of the cellular antioxidant system and one of the most ubiquitous enzymes present in cells [31].

Catalase activity displayed a pattern similar to that of GPx activity (Figure 2f). The primary difference was noted between the 32D BA S and the 32D P line, while the 32D P line showed higher level of catalase activity. The 32D BA S cells had also depressed activity of catalase after exposition to IM when compared to other lines. Interestingly, the comparison of 32D P cells with resistant lines demonstrated a tremendous difference in a basal activity of catalase, which might suggest that the presence of BCR-ABL1 itself may depress activity of this enzyme.

### 3.6. Mitochondrial Membrane Potential Is Lower in IM-Resistant Cells When Compared to IM-Susceptible Cells

In recent years it has been possible to observe an increased focus on the role of mitochondria in carcinogenesis, including what is the role of the alterations in the mitochondrial membrane potential [32].

**Figure 3:**
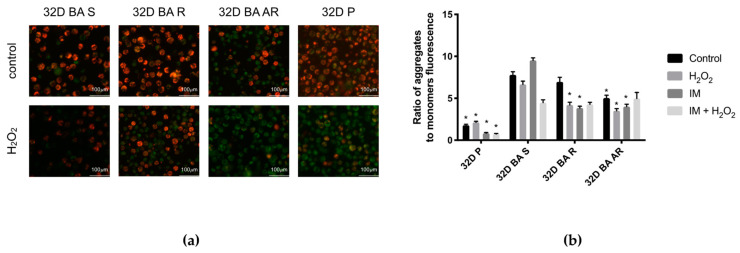
Mitochondrial membrane potential in BCR-ABL1 cells. (**a**) Photos showing leukemic and wild-type, non-cancer cells under different conditions of treatment: control and after 30-min exposure to H_2_O_2_. We stained the cells with cationic dye JC-1 that accumulates in the mitochondrial membrane in a potential-dependent manner. A high potential of the inner mitochondrial membrane leads to the formation of dye aggregates with both excitation and emission shifted towards the red light (530 nm/590 nm), while respective values for JC-1 monomers are 485 nm/538 nm; (**b**) A comparison of the measured ratio in aggregate/monomer ratios of cationic dye JC-1. In BCR-ABL1 positive and parental 32D cells under control conditions and after treatment with IM, H_2_O_2_ or IM + H_2_O_2_. We cultured cells with IM for 24 h and with H_2_O_2_ for 30 min; in case of co-incubation H_2_O_2_ was added at the end of IM incubation. IM was used in 1 μM concentration and H_2_O_2_ in 100 μM. We measured MMP as a ratio of JC-1 monomer to aggregates with a fluorescent plate reader. * *p* < 0.05, the asterisk indicates a significant difference after treatment between a given line and the 32D BA S line, *n* = 4, results shown as mean ± SEM.

A comparison of aggregate/monomer ratios of cationic dye JC-1 under control conditions showed that it was significantly lower in the 32D BA AR line and the 32D P line than in the 32D BA S line. When we exposed cells to IM and H_2_O_2_, we could not see significant differences between 32D BA S and IM-resistant lines (Figure 3b).

## 4. Discussion

The BCR-ABL1 gene leads to genomic instability, which in turn is a major source of resistance of CML cells to TKIs such as IM. However, as recent research shows, how exactly resistant cells avoid cell death in the face of TKIs action is yet to be fully elucidated [16,32]. In this work, we focused on murine 32D cells transformed with the BCR-ABL1 oncogene to create a model of the chronic phase of CML [32]. Some of those cells were transformed with the BCR-ABL1 variant harboring Y253H point mutation, which is a source of resistance to IM, thus making those cells a model of primary resistance to IM. Other cells were cultured in an increasing concentration of IM until they exhibited resistance, thus acting as a model of acquired resistance to IM. As shown previously, such cells form a heterogenous 32D BA AR line, and part of the cell population may derive resistance from point mutations in BCR-ABL1 gene [32]. However, it is important to note that BCR-ABL1-independent mechanisms were also discovered to play role in acquiring resistance to TKI-based therapy [33]. Using 32D cells as a model for CML has an advantage because it allows us to use three different lines to monitor the metabolic disturbances caused by different variants of the BCR-ABL1 gene.

Involvement of ROS in the development of chronic myeloid leukemia has been the focus of research for some time [34]. Earlier research showed elevated levels of ROS production in BCR-ABL1 positive cells when compared to wild-type counterparts [35,36,37]. Additionally, some results indicated that cells with acquired resistance exhibited intrinsically elevated levels of ROS when compared to susceptible cells [38]. It was also observed that measured antioxidant response increases over time in patients undergoing TKI-based chemotherapy and may precede the development of resistance to used chemotherapeutics [39]. On the other hand, it has been recently demonstrated that CML cells resistant to IM through T315I mutation exhibit a lower level of ROS production when compared to CML cells susceptible to IM action. However, those results were gathered using a different cell lines than the ones used by us, which might serve as an explanation for the observed differences [40].

On the other hand, the condition of the continuous oxidative stress in leukemic cells may be interesting therapeutic target, and the possibility of developing redox chemotherapeutics has received much more attention in recent years [41]. Recently it was shown that a pro-oxidant cancer therapy, which selectively killed cancer cells by the ROS production, could augment the conventional chemotherapy [42]. Consequently, the broad extensive oxidative and nitrosative stress triggered by anticancer drugs may intensify genotoxic effects on cancer cells and thus mediate the induction of programmed cell death.

We confirmed that BCR-ABL1-positive cell lines in general have elevated levels of ROS when compared to BCR-ABL1-negative cells, both in control conditions and after exposure to IM and H_2_O_2_. Interestingly, after exposition to IM alone only cells with acquired resistance had significantly lower level of accumulated ROS when compared to 32D BA S cells. This confirms that the mechanism of IM resistance may differ between primary and acquired resistance in the context of the oxidative stress, which was also indicated by our other results.

Earlier studies did not investigate exact changes in ROS metabolism in cells affected by BCR-ABL1 oncogene, neither with reference to enzymes involved in ROS detoxification, nor with relation to IM resistance in CML cells. We focused here on some such systems and hallmarks of ROS activity in resistance to TKIs. There are some signaling molecules e.g., platelet derived growth factor (PDGF) that induces H_2_O_2_-dependent tyrosine phosphorylation in human leukemia cells [37]. Therefore, we decided to use H_2_O_2_ as the external source of free radical production, which can mimic in vivo conditions.

It is worth underlining that in cancer cells, ROS can also play the role of secondary messenger to activate multiple intracellular proteins and enzymes, including the epidermal growth factor receptor, c-Src, p38 mitogen-activated protein kinase, Ras and Akt/protein kinase B [43,44]. Thus, a low level of ROS is also important for keeping the differentiation potential of hematopoietic stem cells [45]. It seems that H_2_O_2_ plays a role as a signaling molecule. The H_2_O_2_ can form from OH-• that removes electrons from surrounding biomolecules and from O2− that spontaneously transform into H_2_O_2_ [46]. However, if the rate of formation of O2−, OH-• and H_2_O_2_is too high they can interact with cell membranes, proteins, and DNA, even damaging it [47].

The results of DNA damage assessment showed that after exposure to IM IM-susceptible cells exhibit higher levels of DNA damage than BCR-ABL1 negative cells. Resistant cell lines seemed to also have more basal DNA damage accumulated. Such an observation is in accordance with an earlier report of BCR-ABL1 self-mutagenesis as a mechanism for developing IM resistance or with recent reports of increased levels of DNA damage in mitochondria in cells resistant to IM action [35,38]. As with ROS accumulation, we demonstrated that exposition to H_2_O_2_ leads to significantly more DNA damage in cells with acquired resistance to IM when compared to 32D BA S, but not in cells with primary resistance, further underlying important differences between those resistances. Exposure to IM leads to an even higher amount of DNA damage in susceptible cells when compared to resistant lines or BCR-ABL1 negative cells. This is compatible with the view that considers DNA damage as one of hallmarks of apoptosis [8]. When undergoing this process, IM susceptible cells accumulate additional DNA damage which, in turn, is one of the factors contributing to rapid cell death [35]. To better understand this situation, we assessed the activity of selected cellular systems involved in maintaining redox homeostasis, i.e., GSH and chosen enzymes in charge of redox balance regulation.

An increase in the GSH level is a known mechanism in the resistance of certain cancer cells to chemotherapeutic agents [48,49,50]. Moreover, a depletion of GSH in cancer cells may make them more susceptible to cell death induction and it can be a molecular target for the novel anti-leukemic agents [51,52]. The results we gathered confirm that GSH levels are higher in resistant and BCR-ABL1 negative cells in comparison to IM-susceptible cells in the face of exposure to IM action. At the same time, they are similar or lower than in susceptible cells under control conditions or after incubation with H_2_O_2_ alone. This suggests that the oncogenic transformation due to the presence of BCR-ABL1 may reduce the physiological levels of GSH and this change is then reversed because of IM resistance development. It is also possible that GSH activation is directly stimulated by IM. This is suggested by the GSH level increase observed in 32D BA R and 32D BA AR cells after IM treatment. This suggests that further investigation into the role of GSH in developing IM resistance is merited.

Selected recent research indicates that cells resistant to IM demonstrate lower levels of expression of ROS scavengers such as GPx and catalase [40]. Interestingly, we observed that after exposure to IM the GPx and catalase activity increased in IM-resistant CML cells in comparison to IM-susceptible cells.

It was previously shown that the induction of apoptosis in leukemic cells led, in some situations, to a decrease in the levels of GSH, which would correspond to our observations of low intracellular GSH concentrations in IM-susceptible cells after exposure to IM [52]. It was also observed that altered GPx expression was present in CML, where the promoter of GPX3 was hypermethylated, which in turn correlated with the lowered expression of GPX3 [53]. Indeed, we observed that the basal activity of GPx under control conditions was higher in 32D P cells in comparison to BCR-ABL1 positive cells, both IM-susceptible and resistant.

We also investigated the activity of catalase and observed it is less active in BCR-ABL1-positive cells when compared to wild-type counterparts. With the reference to the existing literature data, a complicated picture appears here. According to some of the oldest research studies, in both acute and chronic myeloid leukemia there is a substantial increase in catalase activity when compared to normal cells [54,55]. On the other hand, in other forms of cancer such as hepatoma, a lower level of anti-oxidative enzymes, including catalase, can be found [56]. This was used to explain the features of leukemic cells harboring BCR-ABL1 oncogene, which exhibit vulnerability to ascorbate/menadione-induced oxidative stress [57]. On the other hand, authors of selected newer studies pointed out the high expression of catalase in BCR-ABL1 harboring K562 cells [58]. Meanwhile, others indicate that different cell lines might exhibit different patterns of expression and/or activity of catalase [59]. We theorize that depleted catalase levels in 32D BA S cells may be one of the causes of increased oxidative stress, thus contributing to DNA damage and development of acquired resistance. However once resistance is acquired, increase in catalase activity may play a protective role against further excessive, potentially damaging, oxidative stress.

Earlier research showed that a loss of mitochondrial potential is related to the apoptotic death of leukemic cells [60,61]. A research study on the same model used by us revealed lower MMP in resistant cells in comparison to IM-susceptible cells [16]. We confirmed this observing that the 32D BA S line had higher MMP after exposure to IM alone when compared to resistant lines or the 32D P line. This difference was also significant in control conditions in the case of 32D BA AR.

It was theorized that one mode of acquiring resistance to IM takes place through changes in the function of drug transporters that seem to play pivotal role in uptake of several TKIs such as imatinib and dasatinib. Previous research showed an increased expression of human organic cation transporter 1 (hOCT1) correlated with better response to TKI’s treatment in many human leukemia cells [33,62,63]. Moreover, the disturbances in mitochondria homeostasis triggered by IM may be an explanation for our studies that revealed a correlation between IM exposition and changes in MMP.

Overall detected differences in MMP point to the interesting direction of possible mechanisms of IM-resistance. The cellular redox homeostasis status is one of the fundamental factors regulating mitochondrial membrane permeabilization [64]. In our experiments, we used H_2_O_2_, which by the generation of ROS may cause cell death [65]. It was established that oxidative stress, in form of H_2_O_2_ and or other ROS, can activate K+ATP channels and hyperpolarize the plasma membrane potential. This phenomenon can stimulate the mitochondrial ROS production, which may induce the opening of the mitochondrial permeability transition thus decreasing the mitochondrial membrane potential and leading to ATP depletion [66]. Interestingly, mitochondrial DNA (mtDNA) is not packed the same way as chromatin and lacks introns. As a result, mtDNA is more sensitive to oxidative damage than nuclear DNA and there is a higher probability of mutations occurring within coding regions [45]. The increase of mitochondrial ROS production can also reduce the stability of mtDNA and promote DNA damage and mutation that would eventually promote leukemogenesis [67]. These molecular events may happen during our experiments. For instance, in the case of 32D BA S cells, we observed a decrease of MMP after joint exposition to IM and H_2_O_2_. At the same time, it was generally lower in cells resistant to IM or those lacking BCR-ABL1 altogether, when compared to cells sensitive to IM. Thus, further elucidation of role of MMP changes in response to IM, as well as of intrinsic difference in MMP between IM-susceptible and resistant cell lines is needed to carry out.

In this study we investigated some aspects of the connection between the free radicals’ production and the resistance of model CML cells to IM. We showed that the consequence of the free radicals’ production, induced by the incubation of CML murine model cells with H_2_O_2_, is not always augmented by the IM activity. We showed that IM resistant cells exhibit less DNA damage after exposition to IM, which may partially explain their resistance to IM-induced apoptosis. Analysis of the GSH level showed increases in resistant cells, which could further contribute to their resistance, and a similar pattern was observed in the activity of GPx and catalase. Catalase was, in fact, less active in IM-susceptible cells then in wild-type cells, and thus we theorized that increased oxidative stress in IM-susceptible cells could contribute to DNA damage and the development of acquired resistance, but once it was acquired, increase in catalase activity could act against further damaging oxidative stress. Moreover, the measurements of MMP activity suggested a different form of the spontaneous IM-resistance, developed during constant IM treatment like this endured by CML-positive patients.

Our study showed that there are meaningful differences in some aspects of ROS metabolism between susceptible and IM-resistant leukemic cells. However, it also exhibits several limitations that point to future directions in the research. We did not investigate those aspects of ROS metabolism in other leukemic cell lines, nor in cells gathered from patients. We also did not quantify the levels of BCR-ABL1 expression; we only qualitatively correlated its presence with reactions to IM. We believe further analysis of IM expression levels, phosphorylation, or mutational status, including sequencing the cell lines, and activity of ROS scavengers and overall ROS metabolism in leukemic cells is needed to establish more detailed relations between aforementioned factors. Additionally, observed differences between cells with pre-existing and acquired resistance to IM suggest investigation into the role of some other mechanisms of IM-resistance such us alterations in drug transporters activity, as well as verification of the methylation status of genes involved in ROS metabolism. Further research should also focus on the possibility of targeted altering of ROS metabolism in CML cells to facilitate their selective deaths as a therapeutic measure.

## 5. Conclusions

We showed that BCR-ABL1 kinase alters ROS metabolism, and that IM resistance is accompanied by elevated levels of GSH and increased activity of GPx and catalase after treatment with IM. In addition, these differences are associated with alterations in MMP, which are different in cells with primary and acquired resistance. This indicates a direction for future investigation of a possible mechanism for acquiring resistance to TKI.
